# Insights into the Physiology of Childbirth Using Transcriptomics

**DOI:** 10.1371/journal.pmed.0030276

**Published:** 2006-06-13

**Authors:** Roberto Romero, Adi L Tarca, Gerard Tromp

## Abstract

Romero and colleagues discuss a study in *PLoS Medicine* that used microarrays to determine labor-associated gene expression profiles in the human uterus.

Few biological processes as central to the survival of viviparous species are so incompletely understood as childbirth (parturition) [ 1]. Premature labor and prolonged gestation due to failure of labor to commence are associated with an increased risk for perinatal death and long-term handicap. Even when labor begins at term, “failure to progress” is so frequently diagnosed that at least 20% of all births occur through cesarean delivery, the most common operation performed worldwide.

The rationale for performing a cesarean delivery in women whose cervix has failed to dilate in active labor (arrest of dilatation), or in whom the fetal head does not descend after complete dilatation of the cervix (arrest of descent), is that “failure to progress” is an indicator of cephalopelvic disproportion. However, this explanation is unlikely to be true in many cases, because a proportion of mothers with a previous cesarean delivery due to “failure to progress” in labor will deliver a larger baby vaginally in a subsequent pregnancy. Thus, a central question in reproductive biology is: what is “failure to progress” in labor?

A “functional disorder,” rather than cephalopelvic disproportion, may be the underlying reason for a proportion of cesarean deliveries performed after labor has begun. Such “functional disorders” could be due to inadequate preparation of the uterine muscle (myometrium) and/or the cervix for labor. Indeed, accumulating evidence suggests that the myometrium develops a contractile phenotype as pregnancy approaches term and the cervix also undergoes preparatory changes [ 2]. The cellular and biochemical phenomena underpinning preparation of the uterine muscle and the cervix [ 3] are different and have been the focus of intensive investigation [ 4, 5].

## A Molecular Definition of Labor

Scientists and clinicians have tried to develop a molecular definition of labor because there would be substantial clinical implications if “false” labor could be distinguished from “true” labor, both at term and preterm. Just as clinicians, scientists, and patients yearned for a simple method to diagnose pregnancy, so now we aspire to a similar test for labor. But although the identification of active labor is relatively easy, the diagnosis of the onset of labor is difficult but important. Misdiagnosis of labor could have catastrophic consequences for the mother and fetus.

High-dimensional biology techniques (such as transcriptomics, proteomics, metabolomics, and phenomics) have created the expectation of generating a molecular definition of normal or abnormal parturition and, also, identifying biomarkers for these processes [ 1].

A fundamental assumption of transcriptomics is that a comparative study of mRNA expression of a particular tissue will provide information about the biochemical differences between two states (cancer versus no cancer or, in our case, labor versus no labor) and that these differences will provide insight into the physiology and pathology of the condition of interest. Transcriptome signatures have been identified for breast cancer, prostate cancer, and hematologic malignancies. However, using transcriptome signatures to gain insight into physiologic processes remains challenging.

In the case of parturition, several reports have documented that it is possible to identify differentially regulated mRNAs in myometrium, cervix, and chorioamniotic membranes from women not in labor and those in labor using targeted approaches, arrays of cDNAs spotted on membranes (macroarrays), and microarrays [ 2–18]. In general, genes encoding proteins involved in prostaglandin synthesis and in the control of the inflammatory response (chemokines, cytokines, etc.) have been identified as differentially regulated in labor [ 6–21].

## A New Study of Gene Expression in Labor

In a new study published in *PLoS Medicine*, Bukowski et al. determined labor-associated gene expression profiles in the human uterus using microarrays [ 22]. A unique feature of their report is that the investigators sampled three areas of the human uterus in the same women. The uterus is composed of the corpus and the cervix. The corpus is fundamentally a smooth muscle organ (myometrium) responsible for contractions. Two anatomical and functional areas have been identified in the uterine corpus: the fundus and the lower uterine segment. The fundus is thought to contain the uterine pacemakers and generate most of the contractile force of the uterus. The lower uterine segment is believed to relax in labor so that a gradient of force from the fundus to the cervix favors the expulsion of the fetus. The cervix is largely a connective tissue structure, which dilates dramatically during labor to allow the fetus to go through the birth canal.

**Figure 1:**
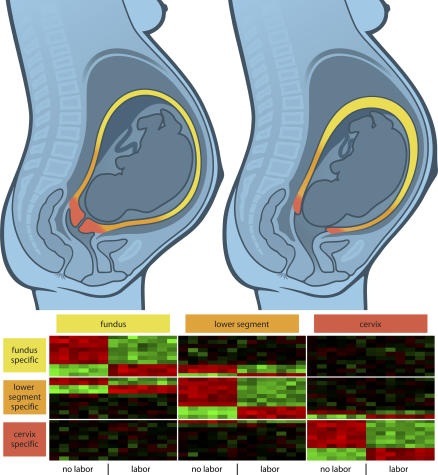
Study of the Transcriptome of the Uterine Fundus, Lower Uterine Segment, and Cervix in Women Not in Labor (Left) and in Labor (Right) This is a diagrammatic representation of the results reported by Bukowski et al. (Illustration: Giovanni Maki)

Bukowski et al. report the results of a study conducted to describe the differences in the transcriptomes of the uterine fundus, lower uterine segment, and cervix before and during labor. Their hypothesis was that labor is associated with changes in the transcriptome in different functional parts of the uterus, and that such stereotypic changes (see Figure 1) are organized into “co-regulated networks.” The researchers obtained samples from the uterine fundus, lower uterine segment, and cervix of six women before the onset of labor and seven women who were in labor. The former group underwent a cesarean section because of abnormal fetal presentation or for having undergone a previous cesarean delivery. Women in labor underwent a cesarean section because of “failure to progress” in labor or “intolerance of labor” (fetal distress). Thus, the experimental design allows examination of two factors: labor status (labor versus no labor) and the topographic uterine component (fundus, lower uterine segment, and cervix). A major strength of the study is that it is the first to have simultaneously examined these three areas of the uterus in the same patient.

The authors concluded that labor results in a change in the transcriptome in each component of the uterus. Moreover, they have provided a list of differentially regulated genes and performed confirmatory studies with quantitative real-time reverse transcriptase polymerase chain reaction (RT-PCR) for two genes: repressor of estrogen receptor activity (REA) and retinoid X receptor alpha (RXR), both of which were down-regulated in the uterine fundus in women in labor. Genes with similar expression profiles were identified, and the authors concluded that networks of co-regulated and co-expressed genes during parturition were discovered.

This report represents a major landmark, as it studied the transcriptome in humans in early labor in three functionally important components of the uterus. The authors are to be commended for depositing the data in the public domain ( http://www.ebi.ac.uk/arrayexpress, accession number E-MEXP-106), thus allowing other investigators to interrogate the dataset and advance the study of labor based upon the contribution of this complex and unique study.

## Our Study of the Transcriptome of the Lower Uterine Segment

One of us (RR) participated in the peer review of Bukowski and colleagues' manuscript and recommended publication to the *PLoS Medicine* editors based upon the richness of the human dataset generated by the authors, the importance of the subject, and the unique nature of the study. Our group has been in the process of conducting a study in which the transcriptome of the lower uterine segment was studied in normal and abnormal labor. We investigated tissues from four different groups: 1) women not in labor at term; 2) women in labor at term; 3) women with an arrest of dilatation; and 4) women with an arrest of descent. Analysis of our as-yet unpublished data ( *n* = 9 or 10 per group) indicated that few changes, if any, were found in the transcriptome of lower uterine segment myometrium between women not in labor and those in early labor (a comparison of group 1 versus group 2). When asked to write this article, we needed to reconcile our observations with those reported in the manuscript by Bukowski et al.

With the permission of the *PLoS Medicine* editors, we contacted Bukowski and colleagues and analyzed the data that the authors had deposited in the public domain. Our analyses differed from those reported in the paper in three ways. First, we employed different criteria to assess suitability of every sample for inclusion in the analysis. The arrays of one patient showed saturation, detected by inspecting the density distribution of probe intensities. The arrays of a second patient were removed because the modal probe intensity was 8-fold higher than all others. Data from a third patient were excluded because they were obtained with a different Affymetrix chip (HG U95A versus HG U95Av2).

Second, we used a different data preprocessing technique, which included robust microarray analysis (RMA) [ 23] to summarize the background corrected, and normalized intensities of the probes into a single value per probe set and array (we also used GCRMA [RMA with background correction modified to account for the content and location of guanosine (G) and cytosine (C) residues in the probe sequences]). Third, a “moderated” *t*-test was used to infer differential expression, and a false discovery rate adjustment of the *p*-value was performed to address the issue of multiple testing. Thus, our analysis is based on a slightly different subset of the data from that used in the original report (five patients in labor and five patients not in labor). However, we have also conducted the analysis using all the arrays from all patients and found similar results to those reported below. The inclusion of the additional arrays rendered the *p*-values less significant.

### Re-Analysis of the Original Data

Bukowski et al. conclude that there is differential spatial regulation of the transcriptome within the human uterus. Our analyses support this conclusion. We also found differences in the transcriptome of the uterine cervix between women in labor and those not in labor using the criteria set forth in the previous paragraph ( Table S1). However, we did not find differences that withstood correction for multiple testing (false discovery rate) in the transcriptome of the uterine fundus between women in labor and not in labor, nor did we find differences in the transcriptome of the lower uterine segment between women in labor and not in labor.

The observation that there is differential expression in the transcriptome among the uterine fundus, the lower segment, and the cervix is reassuring because the tissue composition of the cervix and myometrium is different. Bukowski et al. report that these differences can be detected at the transcriptome level and they are not obscured by the presence of early labor.

Regarding the findings in the myometrium, Bukowski et al. provide the scientific community with a list of the 500 genes with the largest change in expression, and they report that labor was associated with an overall reduction in gene expression in both the uterine fundus and the lower segment (by 71.4% and 72%, respectively). The investigators alert the readers that correction for multiple comparisons will render the differences non-significant. Such is also our conclusion after using a false discovery rate of 5% and a moderated *t*-test. Does this mean that there are no differences in expression for specific genes in the myometrium in women in labor?

Our previous review of the literature in which macroarrays, microarrays, and subtraction hybridization were used to examine differences in gene expression in humans, sheep, rats, and mice indicated that differences could be detected in some circumstances [ 6–10, 13–15, 20]. Moreover, a rich literature documents differential gene expression in the myometrium of women in labor and not in labor when targeted approaches are used, such as Northern blot analysis and quantitative real time RT-PCR for candidate genes implicated in parturition [ 2–5, 17–19, 21]. Thus, a contradiction appears to exist between the results of global, unbiased methods to study the transcriptome and targeted approaches to gene expression.

Two possibilities must be considered to explain this contradiction. First, it is possible that the onset of labor is not associated with a major change in the transcriptome even though there may be differential expression of some specific genes. Transcriptional changes in the myometrium could take place over days or weeks in order to prepare the myometrium to receive a stimulus leading to the onset of labor. Once the contractile phenotype of smooth muscle is acquired, however, major changes may not be necessary for parturition to commence. This would be an example of a physiologic process that may begin without a major and abrupt change in the transcriptome.

Second, global transcriptome analysis is still a recent tool and many challenges remain to be addressed, ranging from optimal hybridization for such a wide number of probes, the appropriate selection of a minimum number of probe sets, and detailed/accurate annotation to optimal statistical analyses. The current platforms may be relatively insensitive for detecting subtle transcriptome changes. Of course, the question of whether or not such subtle changes are biologically important remains open [ 16].

## Conclusion

A fundamental question is whether transcriptome analysis as is currently available can offer valuable information about all physiologic processes. The view of gene expression provided by transcriptome analysis by microarrays is more comprehensive and, perhaps, less biased than that attainable before the “omics” revolution. Yet, to establish if global information about an intermediate product, mRNA, can help us make progress toward understanding physiology is a challenge to those studying transcriptomics.

Global gene expression analysis has been effective for identifying molecular signatures that are diagnostic and prognostic for cancer. Yet, whether current tools will allow us to gain fundamental insights into a physiologic process such as normal parturition remains to be determined.

## Supporting Information

Table S1 Genes Differentially Expressed in Cervix between Labor and Non-Labor Analysis by Romero, Tarca, and Tromp
(22 KB PDF).
