# Supplementary material for: Insights into the Physiology of Childbirth Using Transcriptomics
Source: PLoS Med. 2006 Jun 13;3(6):e276. doi: 10.1371/journal.pmed.0030276 (PMC1475648; doi:10.1371/journal.pmed.0030276)
Supplement: Supplementary file 1 [file pmed.0030276.st001.pdf]

Genes differentially expressed in cervix between labor and non-labor

Gene Symbol	Affy ID	M	A	t	Adjusted P.Val
NDN	36073_at	-0.90	3.09	-6.15	9.33E-03
SC4MOL	33369_at	-0.67	1.78	-6.06	9.33E-03
NPEPPS	39431_at	-0.69	2.12	-6.00	9.33E-03
PTGDS	38406_f_at	-1.72	8.03	-5.33	3.31E-02
CLOCK	36080_at	-0.96	3.86	-5.27	3.31E-02
ECH1	32756_at	-0.94	4.97	-5.25	3.31E-02
HMGN3	37348_s_at	-1.09	3.24	-5.11	3.31E-02
RAMP2	38177_at	-1.14	3.84	-5.07	3.31E-02
CD24	266_s_at	-1.68	2.51	-5.05	3.31E-02
ALDH9A1	33899_at	-1.01	3.30	-5.03	3.31E-02
ADD3	33102_at	-1.74	3.46	-4.98	3.31E-02
TCF12	33348_at	-0.66	2.51	-4.97	3.31E-02
DAAM1	33753_at	-1.38	3.09	-4.97	3.31E-02
DKFZP586A0522	38717_at	-1.26	3.33	-4.86	3.56E-02
CYP3A5	37124_i_at	0.59	2.37	4.86	3.56E-02
PTGDS	216_at	-1.53	8.09	-4.84	3.56E-02
PCNA	1884_s_at	-0.56	2.23	-4.82	3.56E-02
NUMA1	33822_at	-0.55	5.20	-4.82	3.56E-02
MGC5508	39693_at	-1.08	4.97	-4.76	3.70E-02
EIF3S3	35327_at	-0.78	3.69	-4.76	3.70E-02
PTN	234_s_at	-1.05	2.50	-4.73	3.70E-02
CLNS1A	38732_at	-0.58	2.83	-4.72	3.70E-02
SCNN1A	35207_at	-0.94	3.25	-4.71	3.70E-02
PPP1R3C	39366_at	-2.27	2.82	-4.70	3.70E-02
IGF1	1501_at	-1.48	4.71	-4.67	3.70E-02
ALPL	36623_at	0.85	4.05	4.66	3.70E-02
HNRPA0	37334_at	-0.43	1.96	-4.65	3.70E-02
UBE2E3	34850_at	-0.56	2.75	-4.61	3.98E-02
PTN	34820_at	-1.29	3.75	-4.60	3.98E-02
HTR1E	33607_at	0.57	2.58	4.58	3.98E-02
PTPLB	35342_at	-0.87	2.42	-4.57	3.98E-02
DUSP7	39932_at	-0.56	2.55	-4.52	4.48E-02
GNPDA1	34332_at	-0.41	3.87	-4.48	4.75E-02
CIRBP	39864_at	-1.09	4.49	-4.45	5.00E-02
SIAH2	33799_at	-0.72	2.78	-4.44	5.00E-02
